# Supplementary figures and images for: Early Holocene morphological variation in hunter-gatherer hands and feet
Source: PeerJ. 2018 Sep 5;6:e5564. doi: 10.7717/peerj.5564 (PMC6129140; doi:10.7717/peerj.5564)

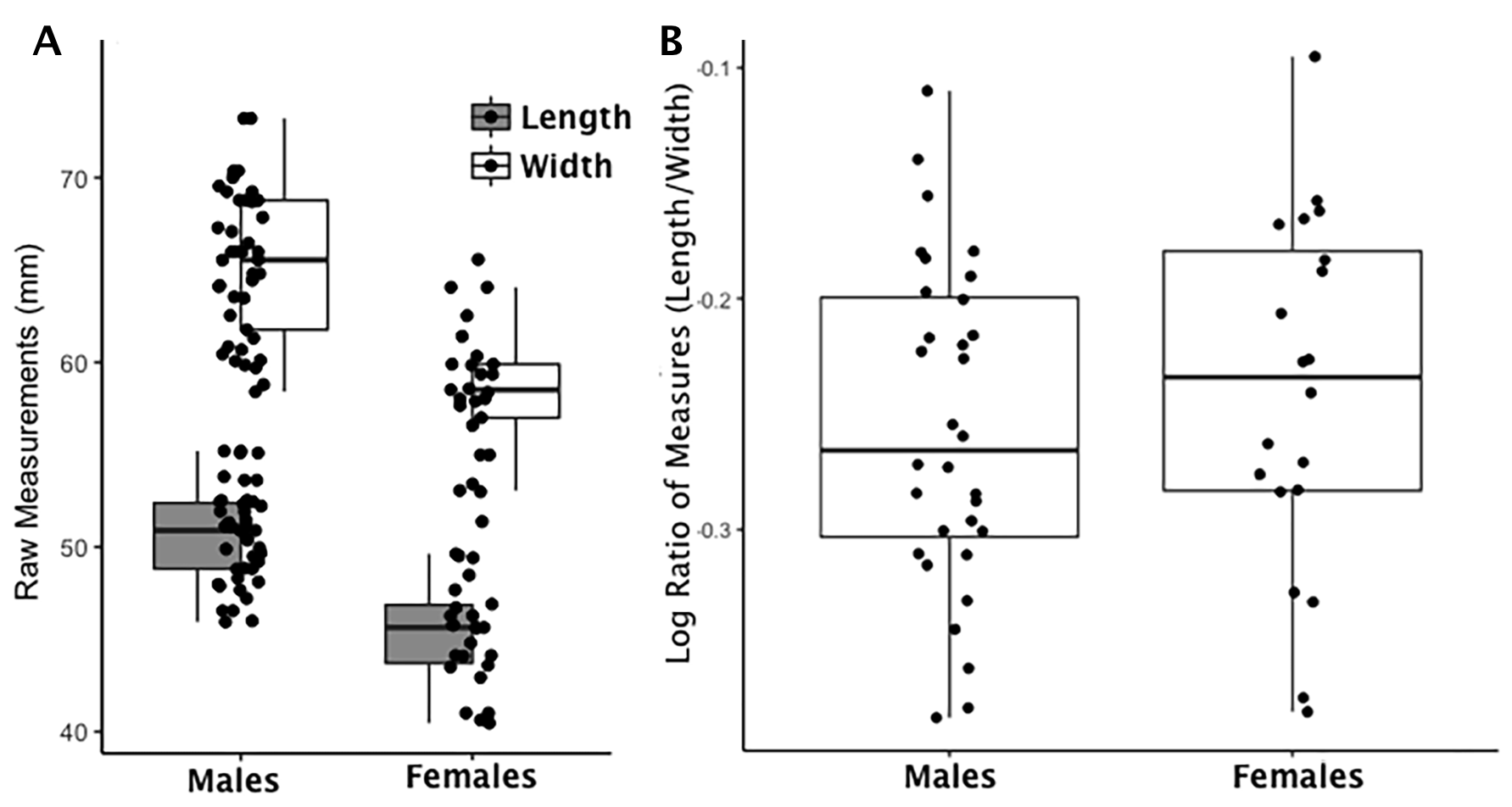

Supplement: Figure S1 — (A) Each data point for the calcaneus load arm represents an individual length (shaded boxplot) or width value (unshaded boxplot); males and females are displayed separately. (B) Each data point for the calcaneus load arm represents an individual logged ratio index value; males and females are displayed separately. [file peerj-06-5564-s001.png]

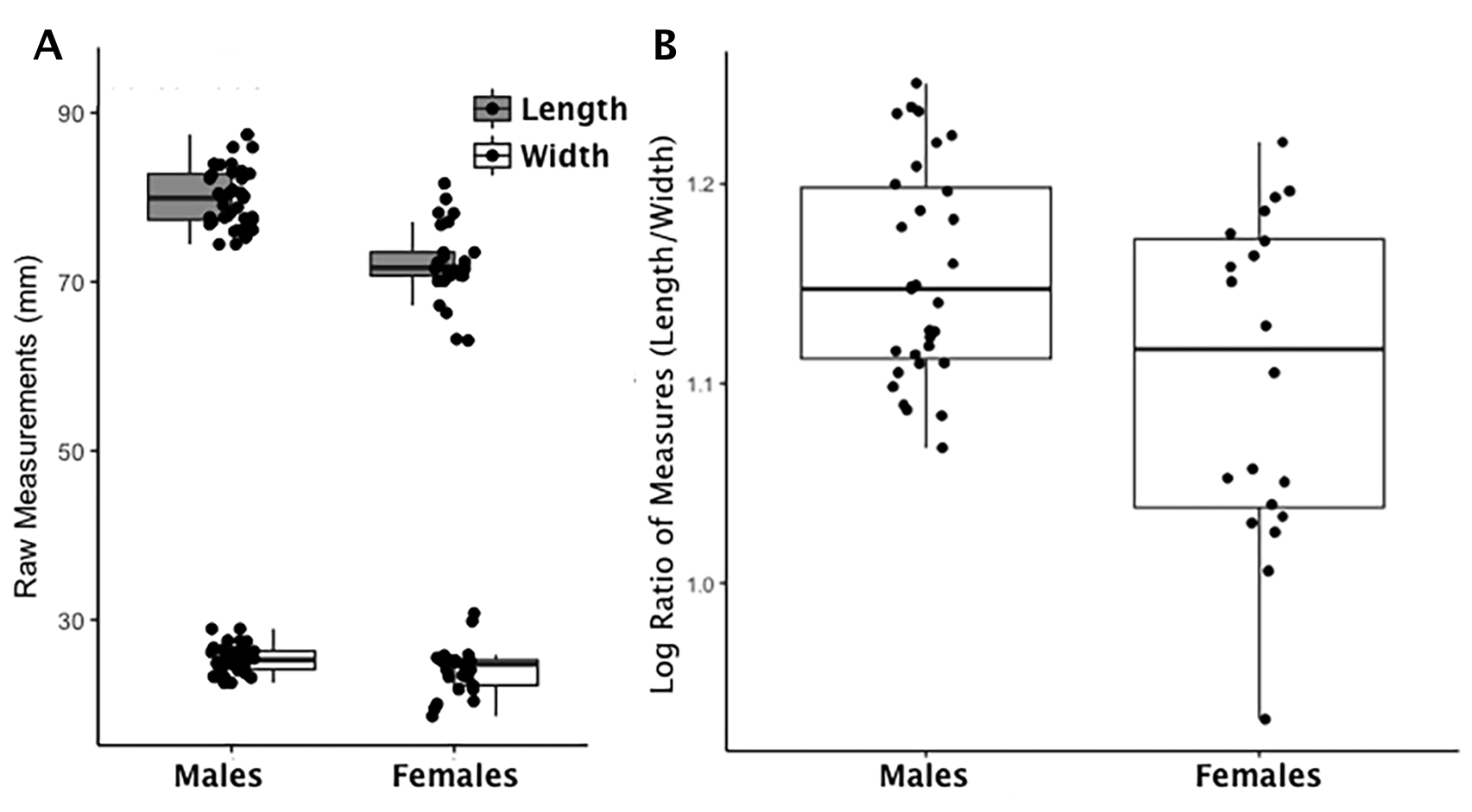

Supplement: Figure S2 — (A) Each data point for the calcaneus represents an individual length (shaded boxplot) or width value (unshaded boxplot); males and females are displayed separately. (B) Each data point for the calcaneus represents an individual logged ratio index value; males and females are displayed separately. [file peerj-06-5564-s002.png]

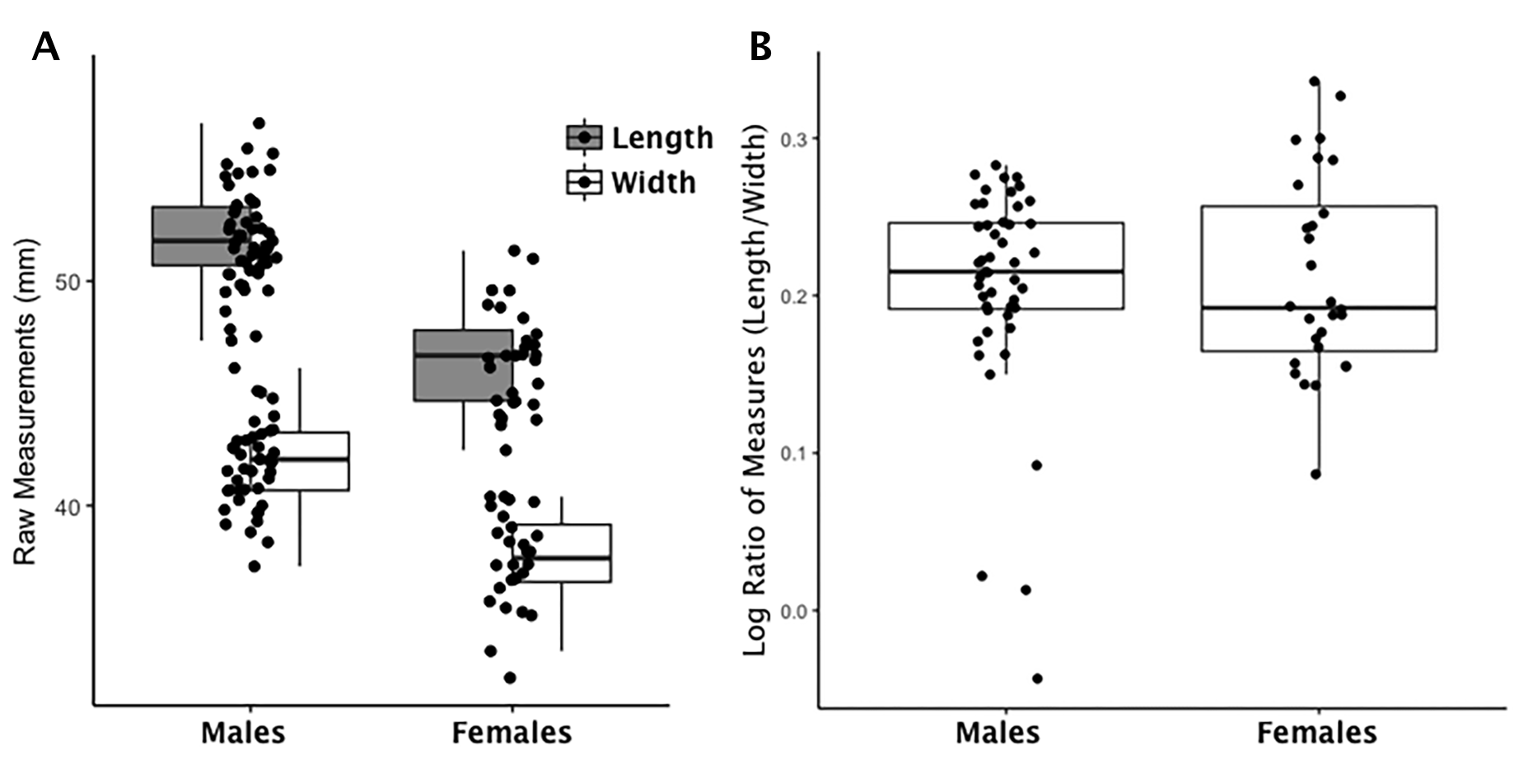

Supplement: Figure S3 — (A) Each data point for the talus represents an individual length (shaded boxplot) or width value (unshaded boxplot); males and females are displayed separately. (B) Each data point for the talus represents an individual logged ratio index value; males and females are displayed separately. [file peerj-06-5564-s003.png]

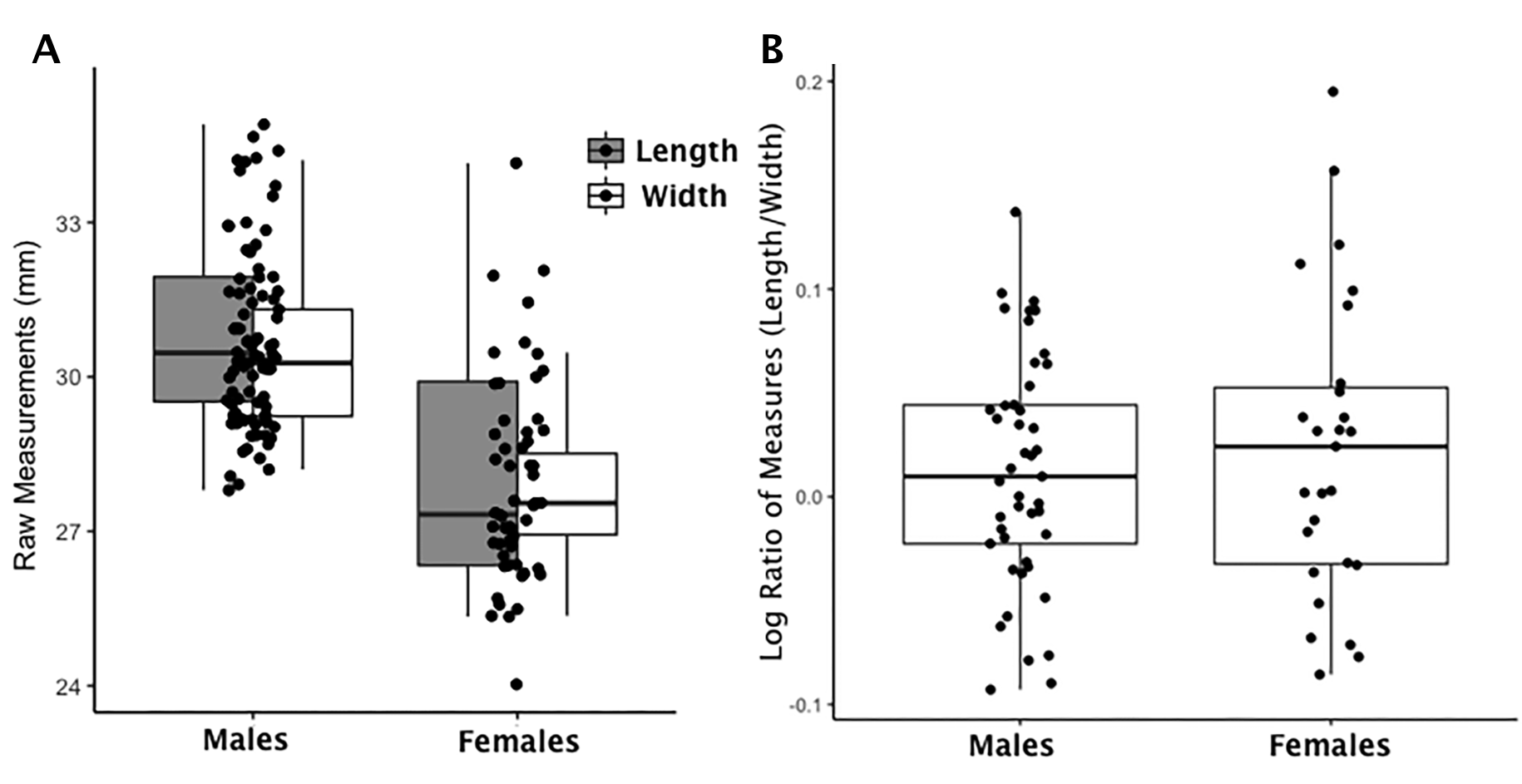

Supplement: Figure S4 — (A) Each data point for the trochlea of the talus represents an individual length (shaded boxplot) or width value (unshaded boxplot); males and females are displayed separately. (B) Each data point for the trochlea of the talus represents an individual logged ratio index value; males and females are displayed separately. [file peerj-06-5564-s004.png]

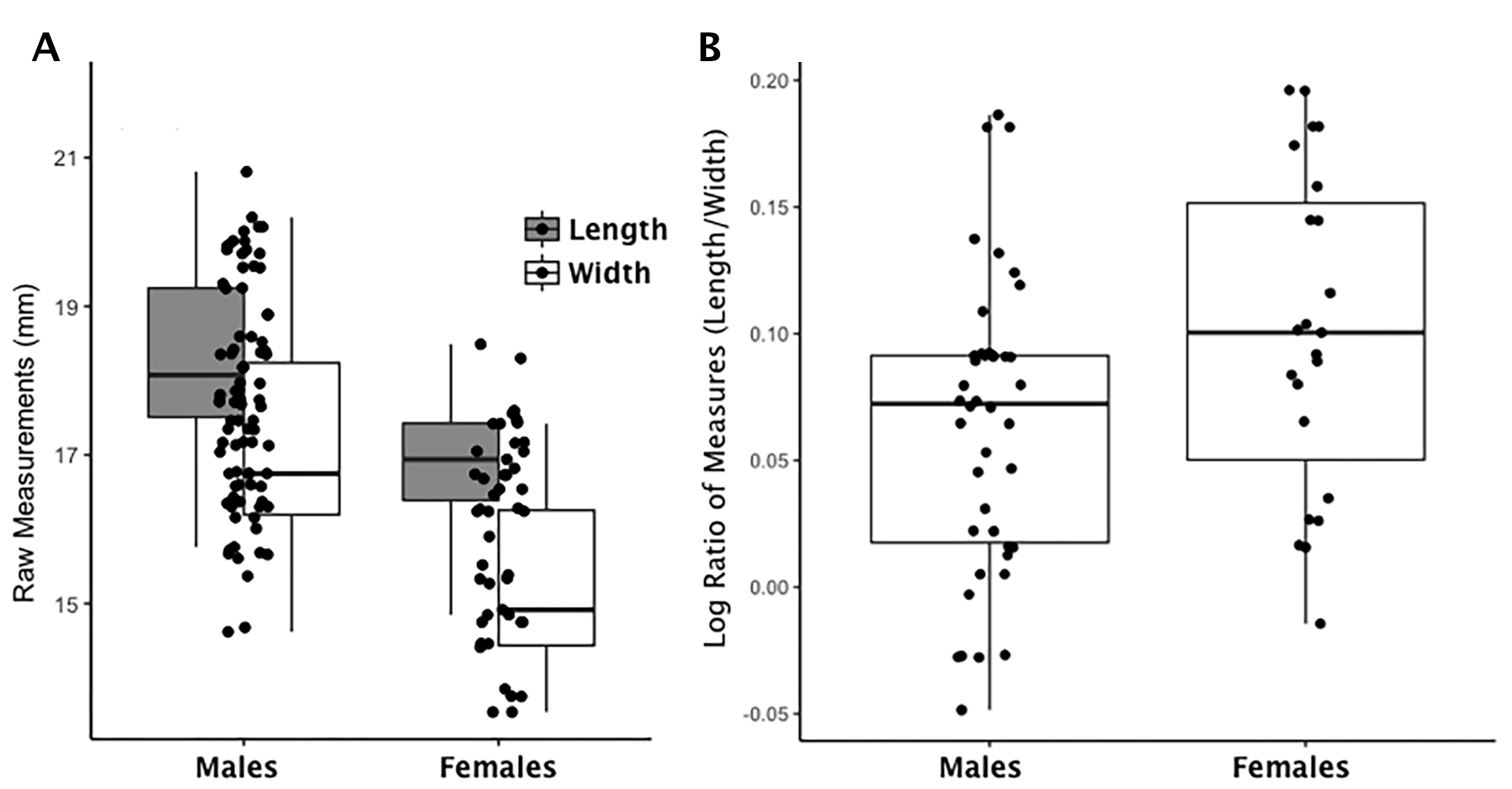

Supplement: Figure S5 — (A) Each data point for the intermediate cuneiform represents an individual length (shaded boxplot) or width value (unshaded boxplot); males and females are displayed separately. (B) Each data point for the intermediate cuneiform represents an individual logged ratio index value; males and females are displayed separately. [file peerj-06-5564-s005.png]

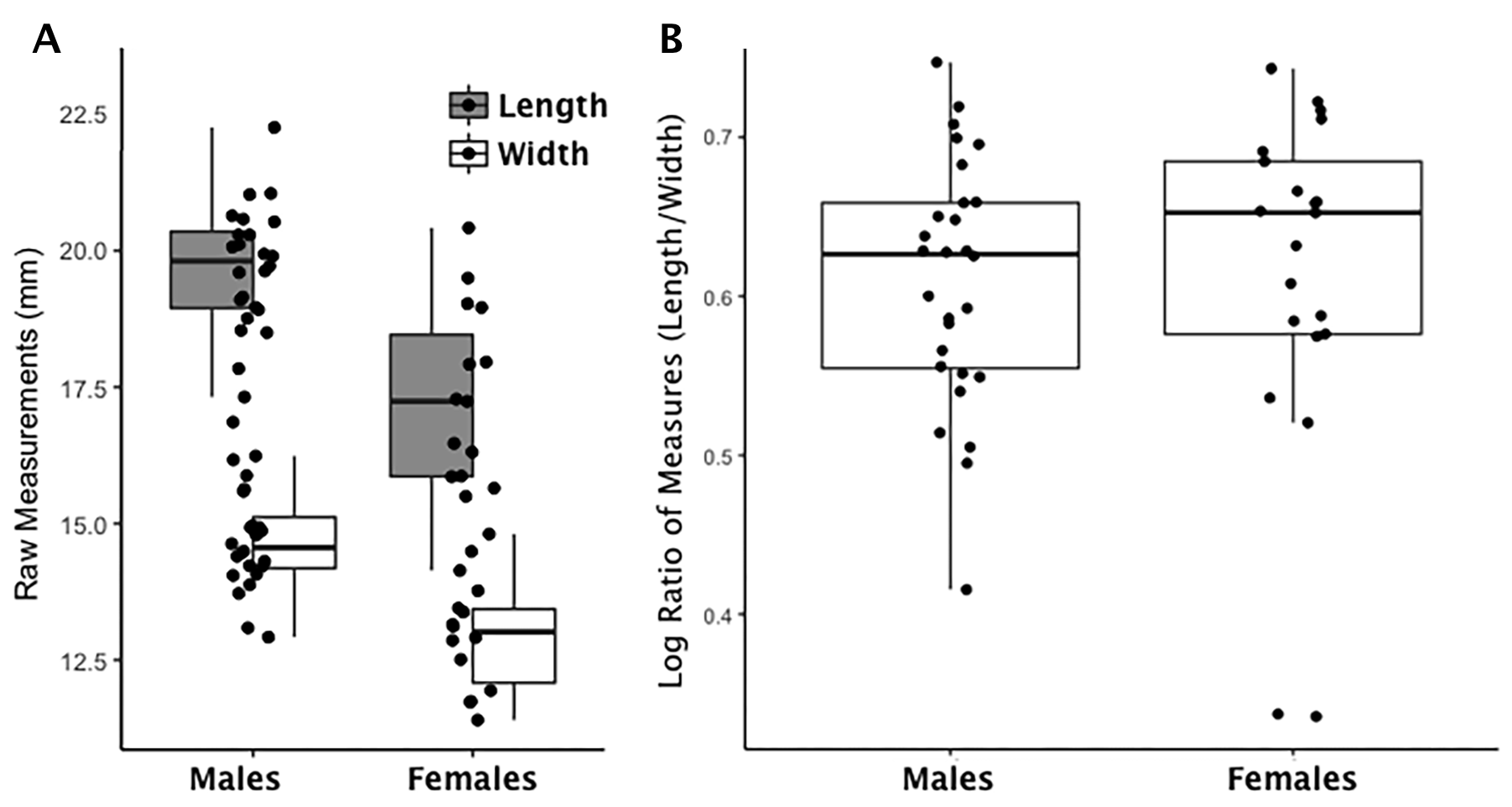

Supplement: Figure S6 — (A) Each data point for the capitate represents an individual length (shaded boxplot) or width value (unshaded boxplot); males and females are displayed separately. (B) Each data point for the capitate represents an individual logged ratio index value; males and females are displayed separately. [file peerj-06-5564-s006.png]

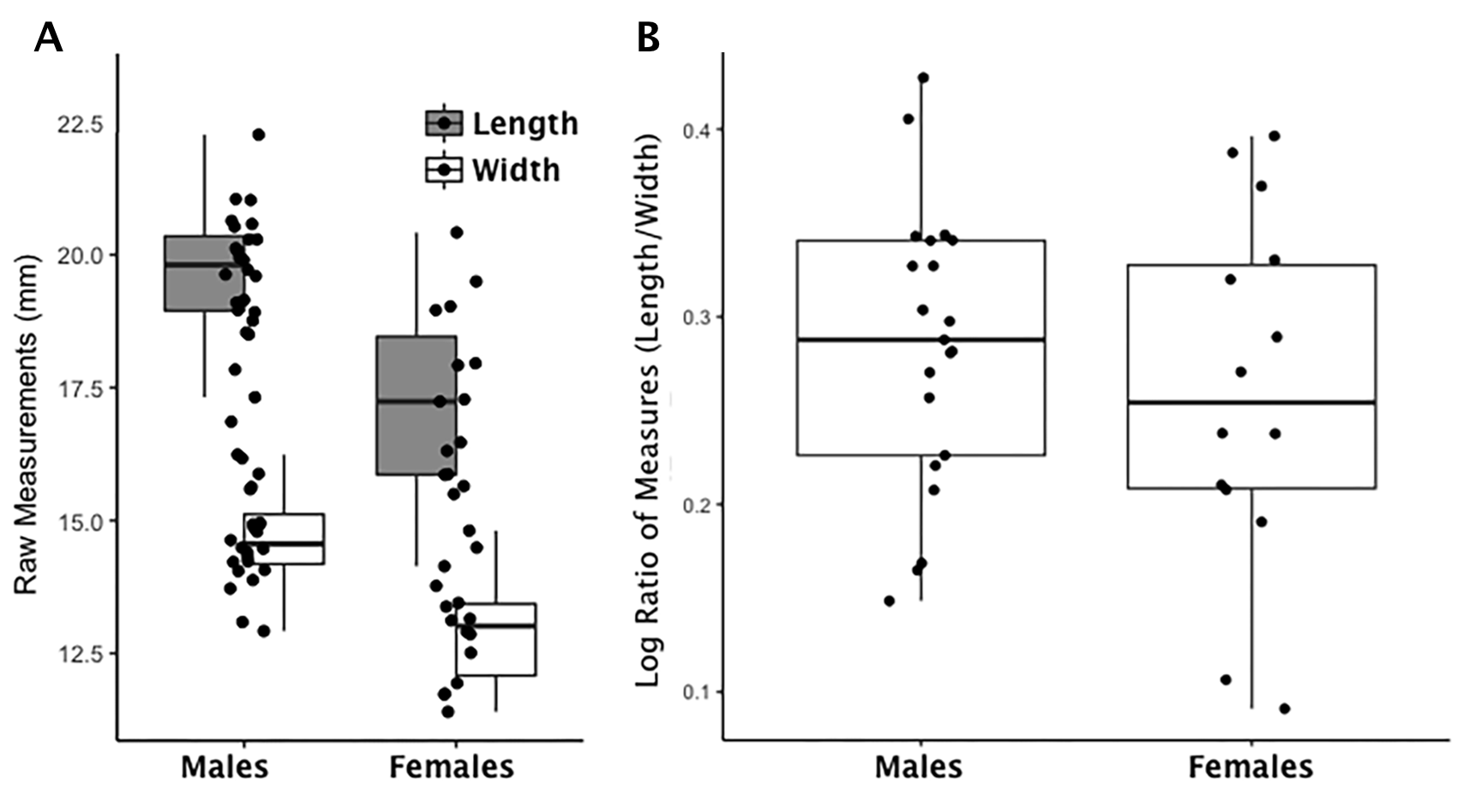

Supplement: Figure S7 — (A) Each data point for the hamate represents an individual length (shaded boxplot) or width value (unshaded boxplot); males and females are displayed separately. (B) Each data point for the hamate represents an individual logged ratio index value; males and females are displayed separately. [file peerj-06-5564-s007.png]

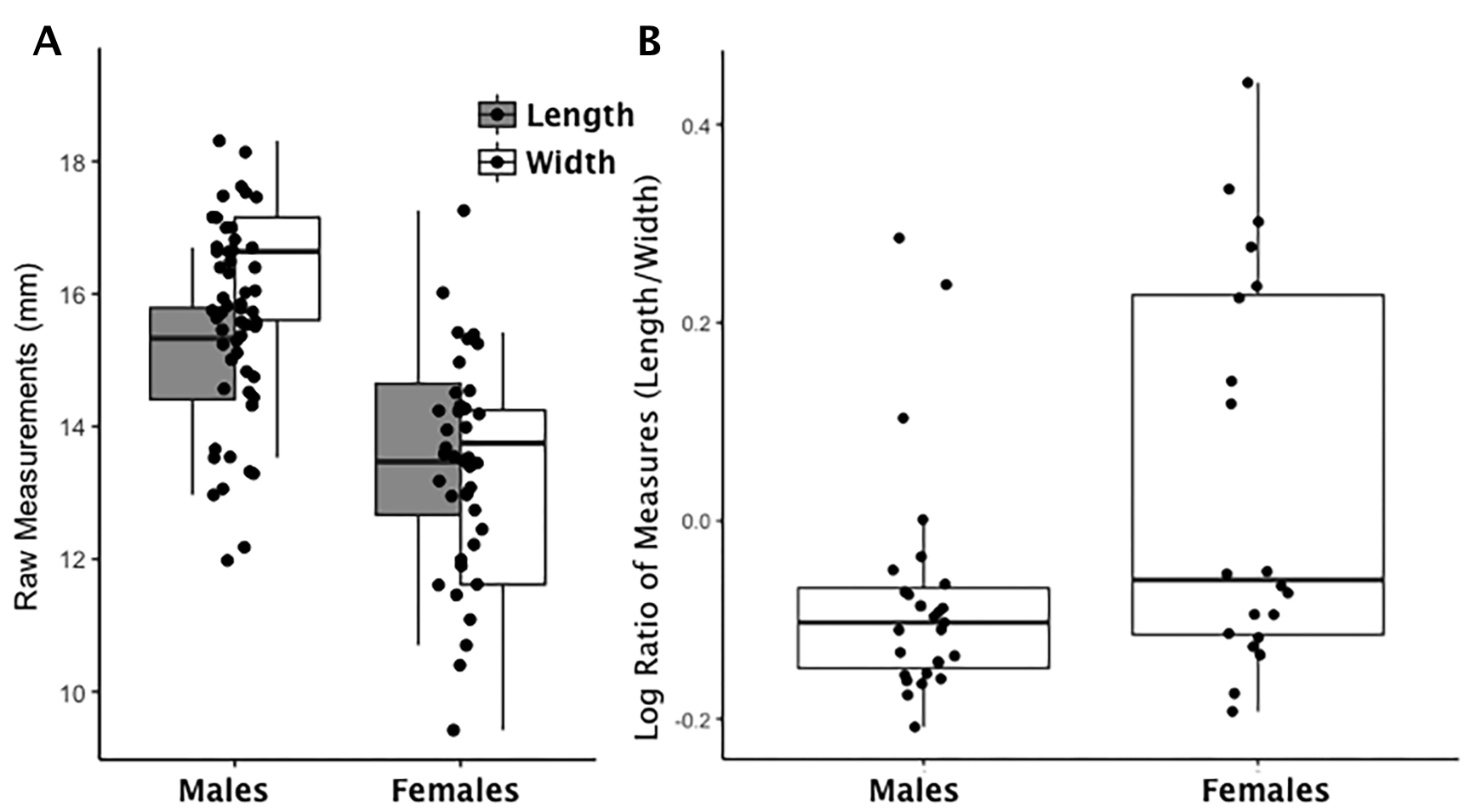

Supplement: Figure S8 — (A) Each data point for the lunate represents an individual length (shaded boxplot) or width value (unshaded boxplot); males and females are displayed separately. (B) Each data point for the lunate represents an individual logged ratio index value; males and females are displayed separately. [file peerj-06-5564-s008.png]

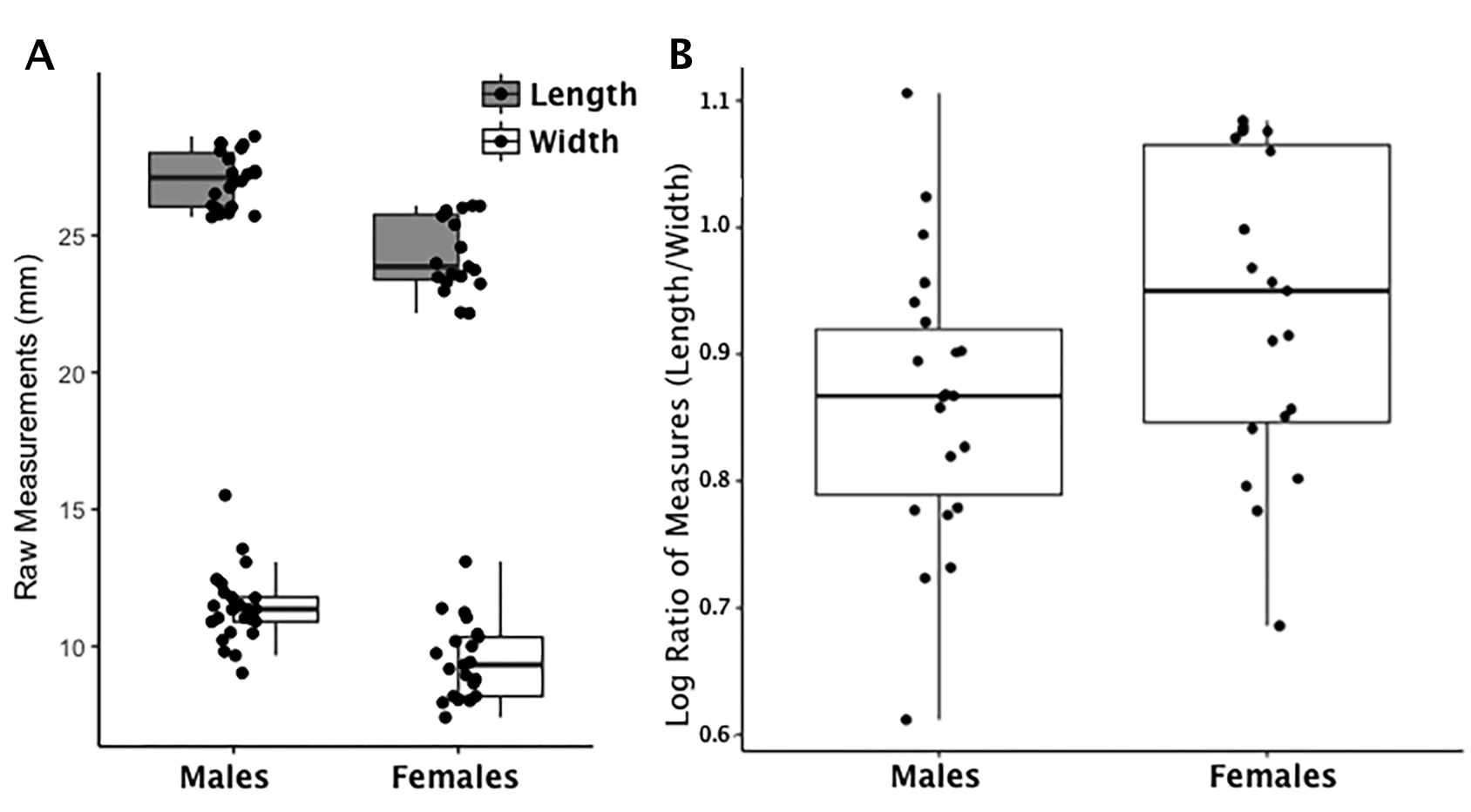

Supplement: Figure S9 — (A) Each data point for the scaphoid represents an individual length (shaded boxplot) or width value (unshaded boxplot); males and females are displayed separately. (B) Each data point for the scaphoid represents an individual logged ratio index value; males and females are displayed separately. [file peerj-06-5564-s009.png]
